# The integrative analysis of competitive endogenous RNA regulatory networks in osteoporosis

**DOI:** 10.1038/s41598-022-13791-0

**Published:** 2022-06-09

**Authors:** Hao Li, Changyuan Wang, Yue Jin, Yuanqing Cai, Huijun Sun, Mozhen Liu

**Affiliations:** 1 Department of Clinical Pharmacology, College of Pharmacy, Dalian Medical University, 9 West Section, Lvshun South Road, Lvshunkou District, Dalian, 116044 China; 2 Department of Orthopaedics, The First Affiliated Hospital, Dalian Medical University, No. 222, Zhongshan Road, Xigang District, Dalian, 116011 China

**Keywords:** Molecular biology, Metabolic disorders, Cell biology, Cell signalling

## Abstract

Osteoporosis (OP) is a common bone disease of old age resulting from the imbalance between bone resorption and bone formation. CircRNAs are a class of endogenous non-coding RNAs (ncRNAs) involved in gene regulation and may play important roles in the development of OP. Here, we aimed to discover the OP‑related circRNA–miRNA–mRNA (ceRNA) network and the potential mechanisms. Six microarray datasets were obtained from the GEO database and the OP‑related differentially expressed genes (DEGs), circRNAs (DECs), and miRNAs (DEMs) were screened out from these datasets. Then, combined with the prediction of the relationships between DEGs, DEMs, and DECs, a ceRNA network containing 7 target circRNAs, 5 target miRNAs, and 38 target genes was constructed. Then the RNA-seq verification by using total RNAs isolated from the femurs of normal and ovariectomized Wistar rats indicated that MFAP5, CAMK2A, and RGS4 in the ceRNA network were closely associated with osteoporosis. Function enrichment analysis indicated that the target circRNAs, miRNAs, and genes were involved in the process of MAPK cascade, hormone stimulus, cadherin binding, rRNA methyltransferase, PI3K-Akt signaling pathway, and Vitamin digestion and absorption, etc. Then a circRNA–miRNA–hub gene subnetwork was constructed and the qRT-PCR analysis of human bone tissues from the femoral head was used to confirm that the transcription of hsa_circR_0028877, hsa_circR_0082916, DIRAS2, CAMK2A, and MAPK4 showed a significant correlation with osteogenic genes. Besides, the two axes of hsa_circR_0028877/hsa-miR-1273f/CAMK2A and hsa_circR_0028877/hsa-miR-1273f/DIRAS2 conformed to be closely associated with OP. Additionally, by constructing a drug-target gene network, RKI-1447, FRAX486, Hyaluronic, and Fostamatinib were identified as therapeutic options for OP. Our study revealed the potential links between circRNAs, miRNAs, and mRNAs in OP, suggesting that the ceRNA mechanism might contribute to the occurrence of OP.

## Introduction

Osteoporosis (OP) is a common geriatric, systemic and metabolic skeletal disease characterized by a general impairment of bone mass and bone strength that results in fragility fractures^1^. With the aging of the population around the world, the incidence of osteoporosis is increasing rapidly and the research on the pathogenesis of osteoporosis and related medicines has become exceedingly needed.

CircRNAs belong to a new class of endogenous non-coding RNAs (ncRNA) with a covalent closed-loop structure and are generated from the linear host genes with a unique ‘back-splicing’ process^2^. CircRNAs are characterized by high tissue specificity and are always with a very low abundance in various tissues compared to the protein-coding mRNAs^3,4^. However, research has shown that circRNAs are rich in miRNA binding sites (miRNA response elements, MREs), which means that circRNAs can act as sponges to combine with miRNAs and compete with mRNAs (Competing endogenous RNA mechanisms, ceRNA)^5,6^. Currently, several studies have shown that circRNAs play important roles in the regulation of many types of diseases via the interaction with disease-associated miRNAs such as neurodegenerative diseases^7^, but the relationship between circRNAs and OP was still dimness. Thus, the study of the mechanisms and roles of OP relative circRNAs will lead to new insight into the basic physiology and disease progression of osteoporosis.

Researchers have identified that some dysregulated circRNAs can influence the development of the skeletal system. For example, Dandan Zhang et al. had shown that circRNA-vgll3 could promote the osteogenic differentiation of adipose-derived mesenchymal stem cells via modulating the miRNA-dependent integrin-α5 expression^8^; Xiqiang Xu et. al had shown that circRNA-0011269 could promote osteoporosis progression through regulation of RUNX2 by combining with miR‐122^9^.

**Figure 1:**
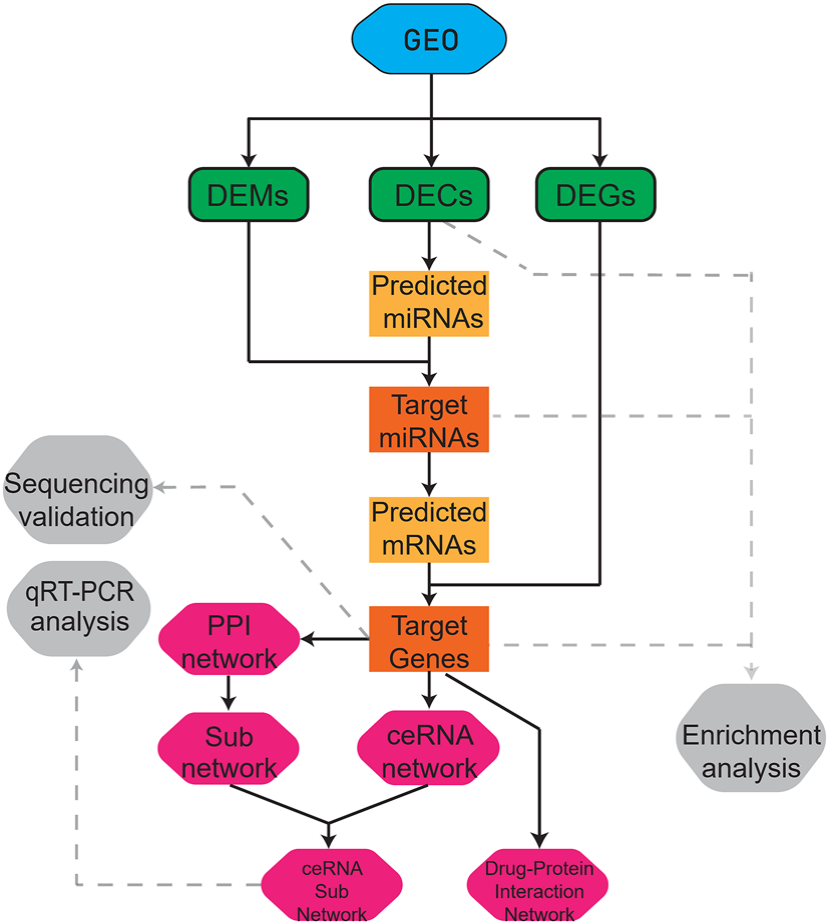
Flowchart of the study. *GEO:* Gene Expression Omnibus, *DEMs:* differentially expressed mRNAs, *DECs:* differentially expressed circRNAs, *DEGs: *differentially expressed genes, *ceRNA:* competitive endogenous RNA, *PPI:* protein–protein interaction network.

In the present study, we collected the expression profiles in osteoporosis patient samples by microarray, and the differentially expressed genes (DEGs), differentially expressed miRNAs (DEMs), and differentially expressed circRNAs (DECs, which also mean the target circRNAs) were identified. Then by the prediction of potential interactions between DEGs, DEMs, and DECs, a ceRNA (circRNA–miRNA–mRNA) regulatory network was constructed. The process flow chart was shown in Fig. 1. To evaluate the main functions of the DECs, DEGs, and DEMs, the gene ontology (GO) annotation and Kyoto Encyclopedia of Genes and Genomes (KEGG) pathway analyses were performed. Besides, RNA-seq analyses were conducted to verify the differential expression of target genes in the ceRNA network. Then a protein–protein interaction (PPI) network of target genes was established and the hub genes which have more effects on the PPI network were extracted; after these, a circRNA–miRNA–hub gene subnetwork which contains less ceRNA axis was also constructed to better understand the pathogenesis of osteoporosis, and the qRT-PCR analysis was carried out to verify the signature of the subnetwork. Besides, we also predicted the OP-associated potential therapeutic drugs from the target genes in the ceRNA network.

In brief, this study attempts to better understand the ceRNA relative pathogenesis of OP and help facilitate the improvement of the diagnosis and therapy of OP.

## Results

### Identification of OP related DEGs, DEMs, and DECs

**Figure 2:**
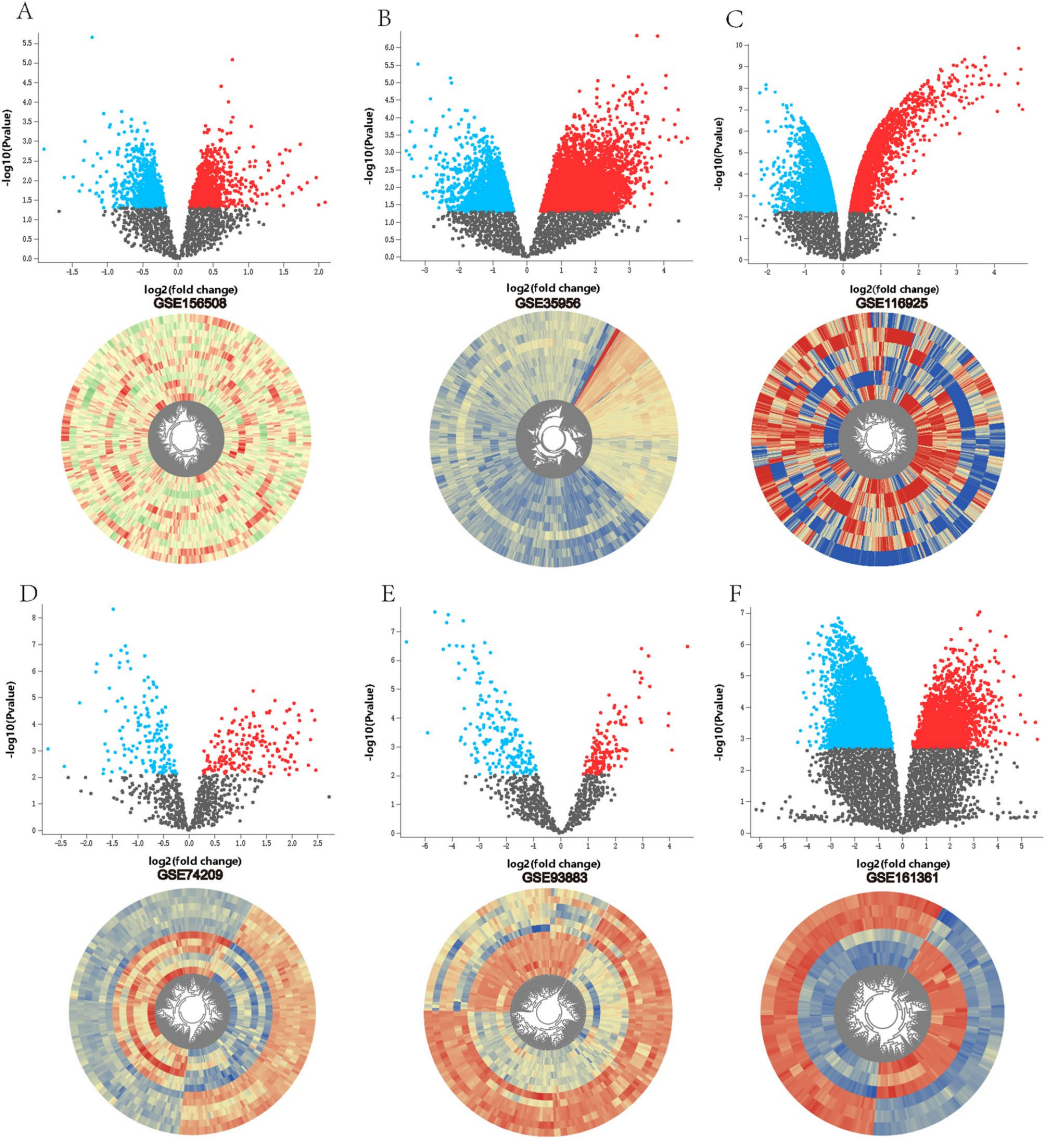
Volcano plots and heatmaps of microarray datasets. (**A**) GSE158508, (**B**) GSE35956, (**C**) GSE116925, (**D**) GSE74209, (**E**) GSE93883, (**F**) GSE161361.

The volcano plots for the DECs, DEMs, and DEGs and the heat maps for the microarray data were shown in Fig. 2. The basic information regarding the expression of circRNAs, miRNAs, and mRNAs in the microarray datasets was listed in Table 1.

**Table 1:** Basic information of the microarray datasets from GEO.

Data source	Type	Platform	First author	Year	Sample source	Sample size (OP/N)	Organism	Region
GSE116925	mRNA	GPL20775	Li C	15-08-2018	BMSCs	3/3	Mus musculus	China
GSE35956	mRNA	GPL570	Benisch P	25-03-2019	BMSCs	5/5	Human	Germany
GSE156508	mRNA	GPL16686	Panach L	08-10-2020	Primary osteoblasts	6/6	Human	Spain
GSE74209	miRNA	GPL20999	de Ugarte L	22-12-2015	Femoral neck trabecular bone	6/6	Human	Spain
GSE93883	miRNA	GPL18058	Sun M	13-01-2020	Plasma	6/6	Human	China
GSE161361	ciRNA	GPL28148	Hua F	11-03-2021	Serum	3/3	Human	China

*GEO* gene expression omnibus, *OP osteoporosis*, *N* normal.

**Figure 3:**
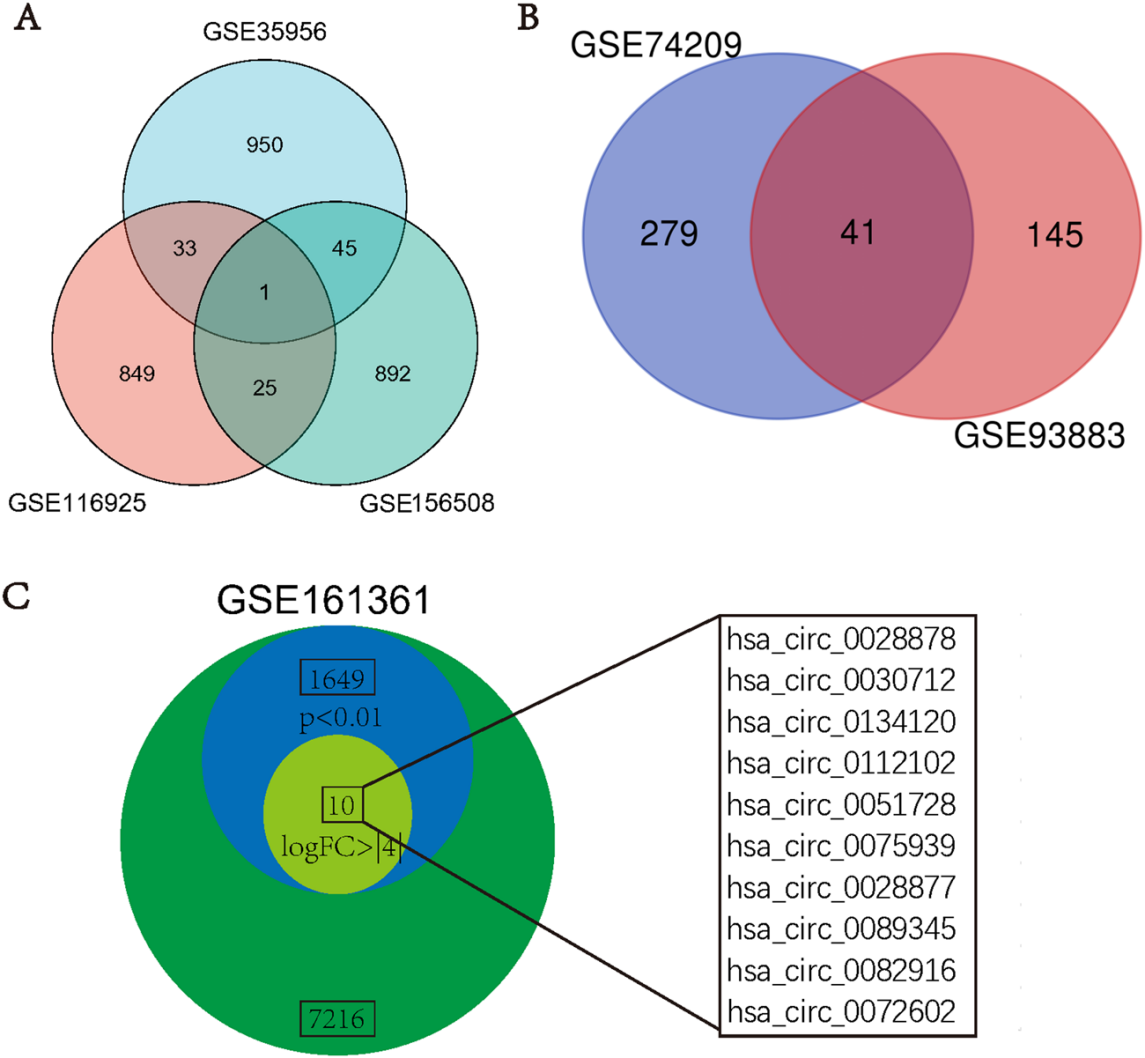
Identification of DEGs, DEMs, and DECs. (**A**) 104 DEGs were obtained from three GEO data sets, (**B**) 41 DEMs were obtained from three GEO data sets, (**C**) 10 DECs (also means target circRNAs) were obtained from GSE161361. DEGs, differentially expressed genes. DEMs, differentially expressed miRNAs. *DECs* differentially expressed circRNAs.

Our study included 3 microarray datasets (GSE116925, GSE35956, and GSE156508) to obtain the DEGs. After the analysis with the GEO2R tool, we set the cut-off point as an adjusted p-value < 0.05 and the absolute value of log FC > 1. Then the mRNAs were ranked by the absolute value of log FC. After that, the top 1000 mRNAs were picked out as the candidate mRNAs. There were 988 mRNAs found in the GSE35956 data set, 992 mRNAs found in the GSE156508 data set, and 908 mRNAs found in the GSE116925 data set after the duplicate items were removed. Subsequently, we integrated the filtered mRNAs of the three datasets and integrated them with a Venn diagram, and finally, 104 mRNAs could be found in at least two microarray data sets and were defined as the DEGs (Fig. 3A).

As for the miRNAs, there were 320 miRNAs found in the GSE74209 data set and 186 miRNAs found in the GSE93883 data set when the cut-off point was set as adjusted p-value < 0.05 and the absolute value of log FC > 1; then finally the 41 overlapped miRNAs were defined as the DEMs (Fig. 3B).

As for the circRNAs, we got the top 10 differentially expressed circRNAs (DECs) from the dataset GSE161361 data set when the cut-off point was set as adjusted p value < 0.05 and absolute value of log FC > 1. The circRNAs were ranked by the absolute value of log FC. Then the top 10 DECs were defined as the DECs (Fig. 3C, which also mean the target circRNAs), which might involve the OP-associated ceRNA mechanisms. The basic information regarding the DECs was listed in Table 2.

**Table 2:** Basic information of the 10 target circRNAs.

Circbase.ID	Adj. P val	P value	logFC	Chromosome	circStart	circEnd	Host gene
hsa_circ_0028878	0.00254	5.43E−07	4.333819	chr12	120,878,256.00	120,878,529.00	COX6A1
hsa_circ_0030712	0.00358	7.18E−06	4.235667	chr13	99,512,658.00	99,540,801.00	DOCK9
hsa_circ_0134120	0.0042	1.05E−05	4.672547	chr7	31,904,555.00	31,961,207.00	PDE1C
hsa_circ_0112102	0.00486	1.47E−05	4.026275	chr1	222,838,847.00	222,839,203.00	MIA3
hsa_circ_0051728	0.00662	3.36E−05	− 4.14181	chr19	48,636,238.00	48,660,397.00	LIG1
hsa_circ_0075939	0.00712	4.08E−05	4.938744	chr6	31,752,371.00	31,752,491.00	VARS
hsa_circ_0028877	0.00769	4.96E−05	4.144324	chr12	120,876,181.00	120,878,529.00	COX6A1
hsa_circ_0089345	0.00772	5.00E−05	− 4.04768	chr9	136,216,766.00	136,217,582.00	RPL7A
hsa_circ_0082916	0.00823	5.85E−05	4.05382	chr7	150,773,712.00	150,777,881.00	FASTK
hsa_circ_0072602	0.00955	8.41E−05	4.184512	chr5	61,689,781.00	61,690,434.00	DIMT1

### Identification of the target miRNAs and target genes and construction of the circRNA–miRNA–mRNA network

**Figure 4:**
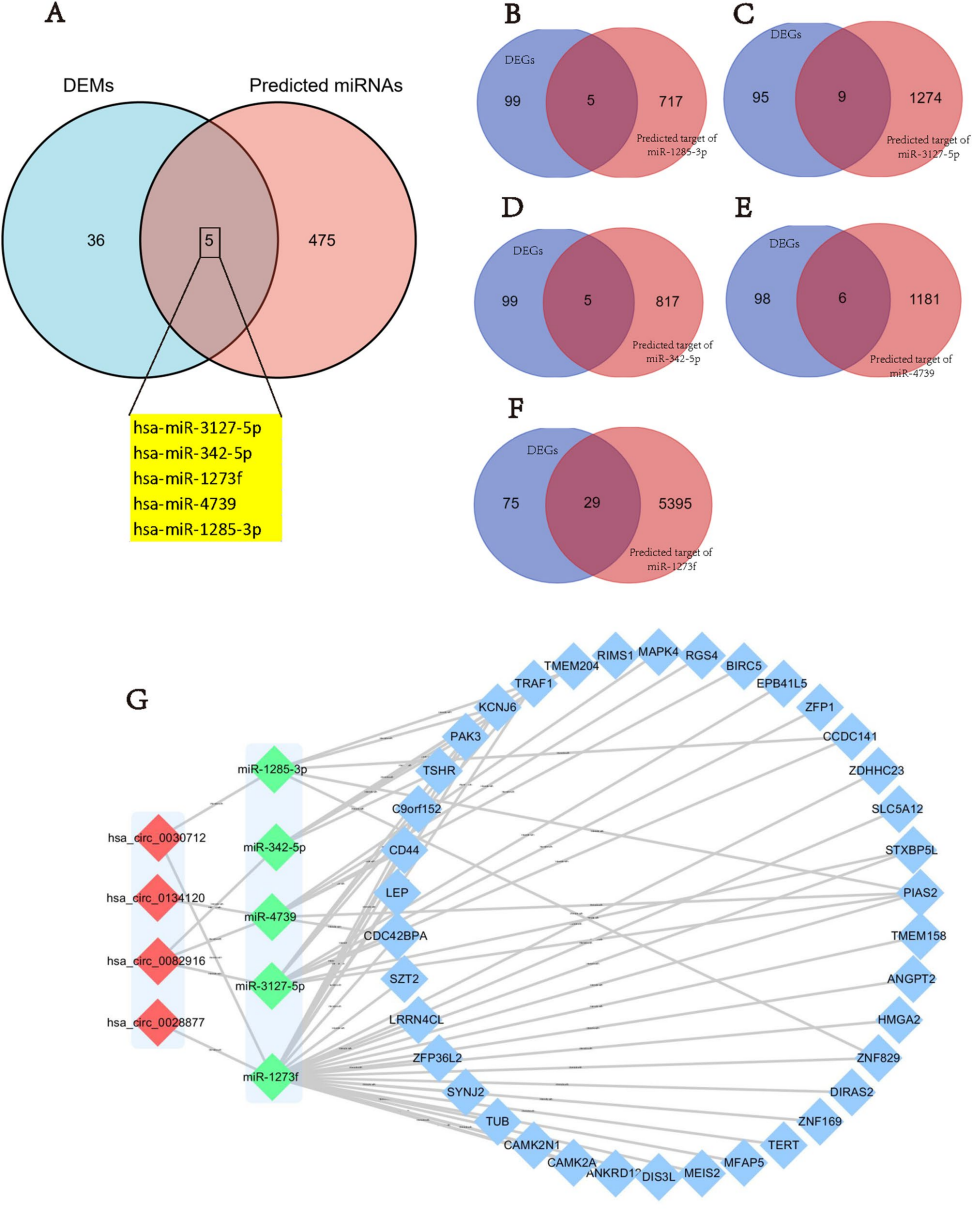
Identification of the target miRNAs and target genes and construction of the circRNA–miRNA–mRNA network. (**A**) Identification of the target miRNAs, (**B**–**F**) Identification of the target genes by Venn analysis, (**G**) CircRNA–miRNA–mRNA network.

By intersecting the target circRNAs’ predicted target miRNAs and DEMs obtained from the GEO database, we confirmed 5 OP-associated target miRNAs finally (Fig. 4A).

The predicted mRNAs which could bind to the target miRNAs were obtained by using the miRWalk and TargetScan websites. Then by intersecting the predicted mRNAs and DEGs obtained from GEO, 38 target genes were confirmed after the duplicates were removed (Fig. 4B–F).

**Figure 5:**
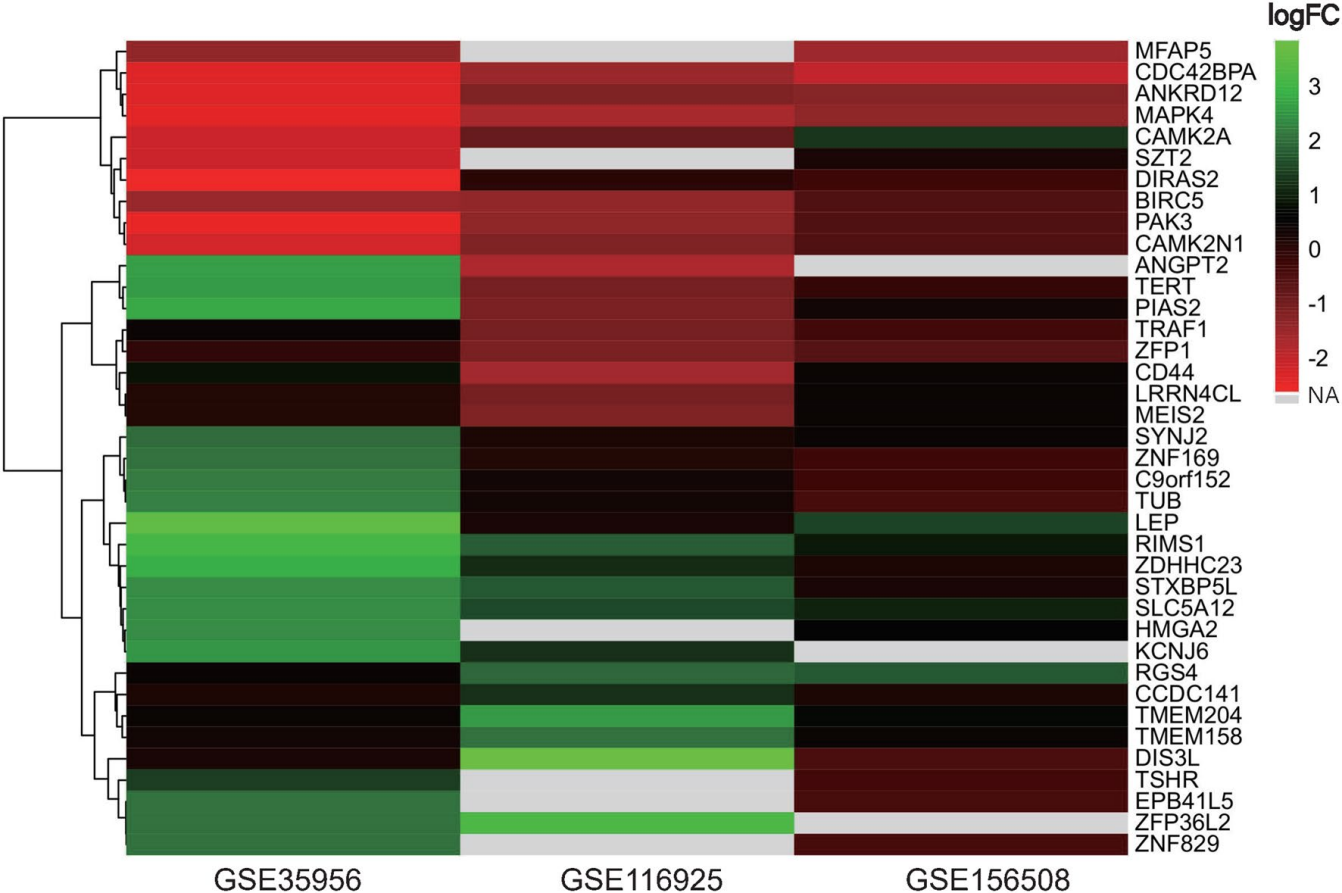
Heatmap for the 38 target genes in the circRNA–miRNA–mRNA network by GEO data sets.

Finally, the circRNA–miRNA–mRNA network containing circRNA-miRNA pairs and miRNA-mRNA pairs was generated by the Cytoscape software (Fig. 4G), and the heatmap of the 38 target genes included in the network was constructed based on the expression difference of the genes in the GEO database (Fig. 5).

### Function enrichment analysis of target genes, target miRNAs, and DECs

**Figure 6:**
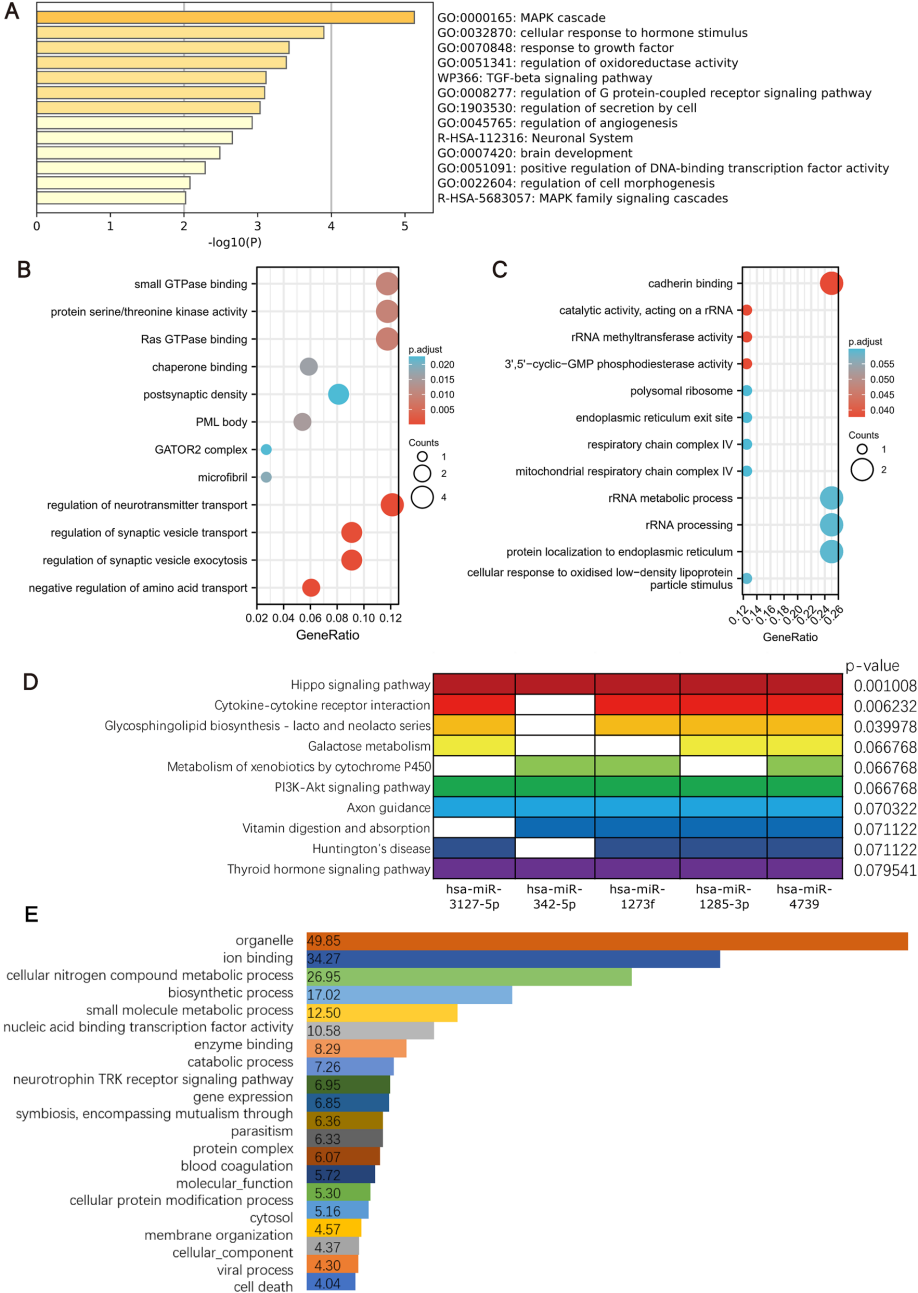
Enrichment analysis of target genes, target miRNAs, and DECs. (**A**, **B**) Enrichment analysis of the target genes. (**C**) Enrichment analysis of the host genes of target circRNAs. (**D**, **E**) Enrichment analysis of the target miRNAs.

The function enrichment analysis of target genes was performed by the Metascape website and the R package of cluster profile combine with the ggplot2 package (version 3.3.3). As shown in Fig. 6A and B, the enrichment analysis of the target genes was mainly enriched in the osteoporosis-related ‘MAPK cascade’^10^, ‘cellular response to hormone stimulus’^11^, and ‘regulation of oxidoreductase activity’^12^, etc. which revealed that the target genes might affect the development of osteoporosis in these signal pathways.

Because the circRNAs belong to a type of novel non-coding RNA, their function was still not understood well and the functional analysis could not be directly performed on the DECs. Thus, we hypothesized that the host genes of DECs which transcribe the precursor RNAs of circRNAs, play significant roles in the ceRNA network and the analysis of the host genes could reveal the potential mechanisms of DECs. Then the ggplot2 package and clusterProfiler were applied for further enrichment analysis. As shown in Fig. 6C, the enrichment analysis of the host genes was mainly enriched in the osteoporosis-related items of ‘cadherin binding’^13^, ‘rRNA methyltransferase activity’^14^, and ‘cellular response to oxidized low-density lipoprotein’^15^, et.al.

The enrichment analysis of the target miRNAs was performed by the mirPath v.3 tool on the DIANA Tools website. MiRPath is a miRNA pathway analysis web-server in which the enrichment analysis of miRNAs was performed indirectly through the corresponding target genes^16^. As shown in Fig. 6D and E, the enrichment analysis results were mainly enriched in the osteoporosis-related items of ‘PI3K-Akt signaling pathway’^17^, ‘Vitamin digestion and absorption’^18^, ‘Thyroid hormone signaling pathway’, and ‘cell death’^11^, etc.

The above enrichment analysis results are all OP-related items, and this indicated that the target genes, target miRNAs, and DECs are closely related to OP.

### RNA-seq verification of target genes

**Figure 7:**
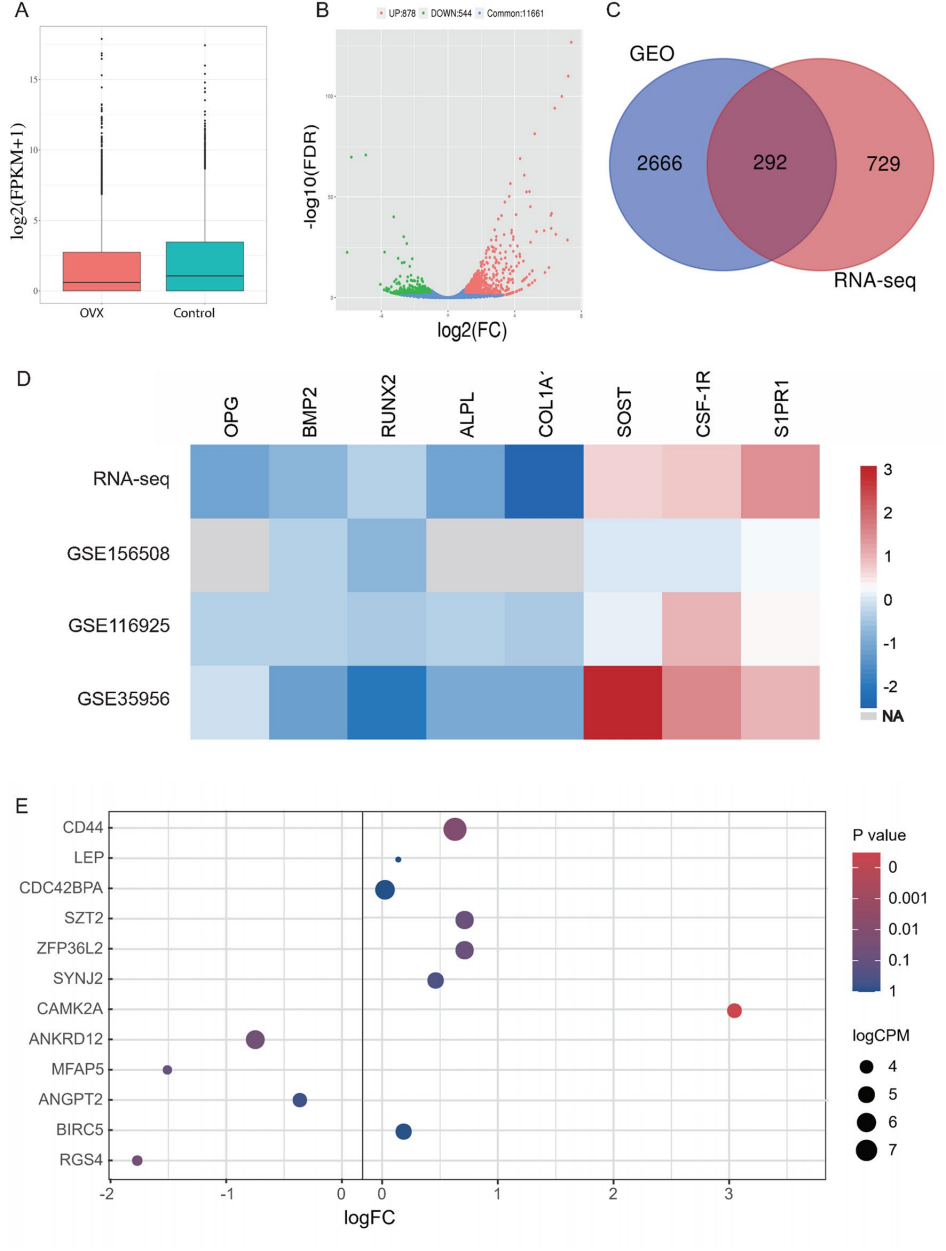
RNA-seq verification of target genes. (**A**) Comparison of the FPKM distribution of all samples. (**B**) Volcano Plot of gene expression differences. (**C**) RNA-seq data and GEO data were compared by a Venn diagram. (**D**) Comparisons of OP relative genes in GEO databases and RNA-seq data. (**E**) Bubble chart of verification of target genes by RNA-seq data.

To further analyze the relationships between the target genes and OP and to verify the reliability of DEGs derived from the public database, a model of osteoporosis in ovariectomized rats was established and the RNA-seq was proceeded to analyze the differentially expressed genes between the osteoporosis rats and the normal rats (Fig. 7A). To find differentially expressed mRNAs, the selection criteria were as follows: (1) mRNA was detected among all samples, (2) Absolute value of log FC > 1, and (3) P < 0.05 according to the t-test. The results showed that there are 1422 mRNAs differentially expressed between the two groups (Fig. 7B), and there were 878 up-regulated mRNAs and 544 down-regulated genes in the osteoporosis group compared with the normal group (Data not shown). Besides, by comparing the differentially expressed mRNAs from RNA-seq and GEO database, we found that about 30% of the differentially expressed mRNAs obtained from the RNA-seq also existed in the GEO database after the duplicate mRNAs were removed (Fig. 7C); and there were 12 overlapped genes found between the 104 DEGs and the RNA-seq data obtained differentially expressed mRNAs, such as CAMK2A and DCD42BPA (Supplement Fig. 3). Comparison of GEO databases and RNA-seq data showed that the expression of OP relative indicator genes, such as OPG, BMP-2, RUNX2, CSF-1R, etc. were all changed obviously (Fig. 7D), which showed the data reproducibility between the GEO databases and RNA-seq data. And as shown in Fig. 7E, in the RNA-seq data, the gene expression levels of MFAP5, CAMK2A, and RGS4, etc., which were target genes of the ceRNA network (Fig. 4G), were significantly different between the control and OVX rats according to the log FC values. Besides, GO and KEGG enrichment analysis of the DEGs obtained from our RNA-seq data indicated that the down-regulated genes in the OVX rats were primarily enriched in the PI3K-Akt signaling pathway, endocrine resistance pathway, ‘osteoblast differentiation’, and ‘bone development’, etc. (Supplement Fig. 2A and B), while the up-regulated genes in the OVX rats were primarily enriched in the thyroid hormone signaling pathway and HIF-1 signaling pathway, etc. (Supplement Fig. 2C and D), which are all the OP relative items. The above results indicated that the RNA-seq derived DEGs and GEO datasets derived DEGs were in good agreement and the target genes MFAP5, CAMK2A, and RGS4 were closely related to OP.

### PPI network establishment and hub genes identification of the target genes

**Figure 8:**
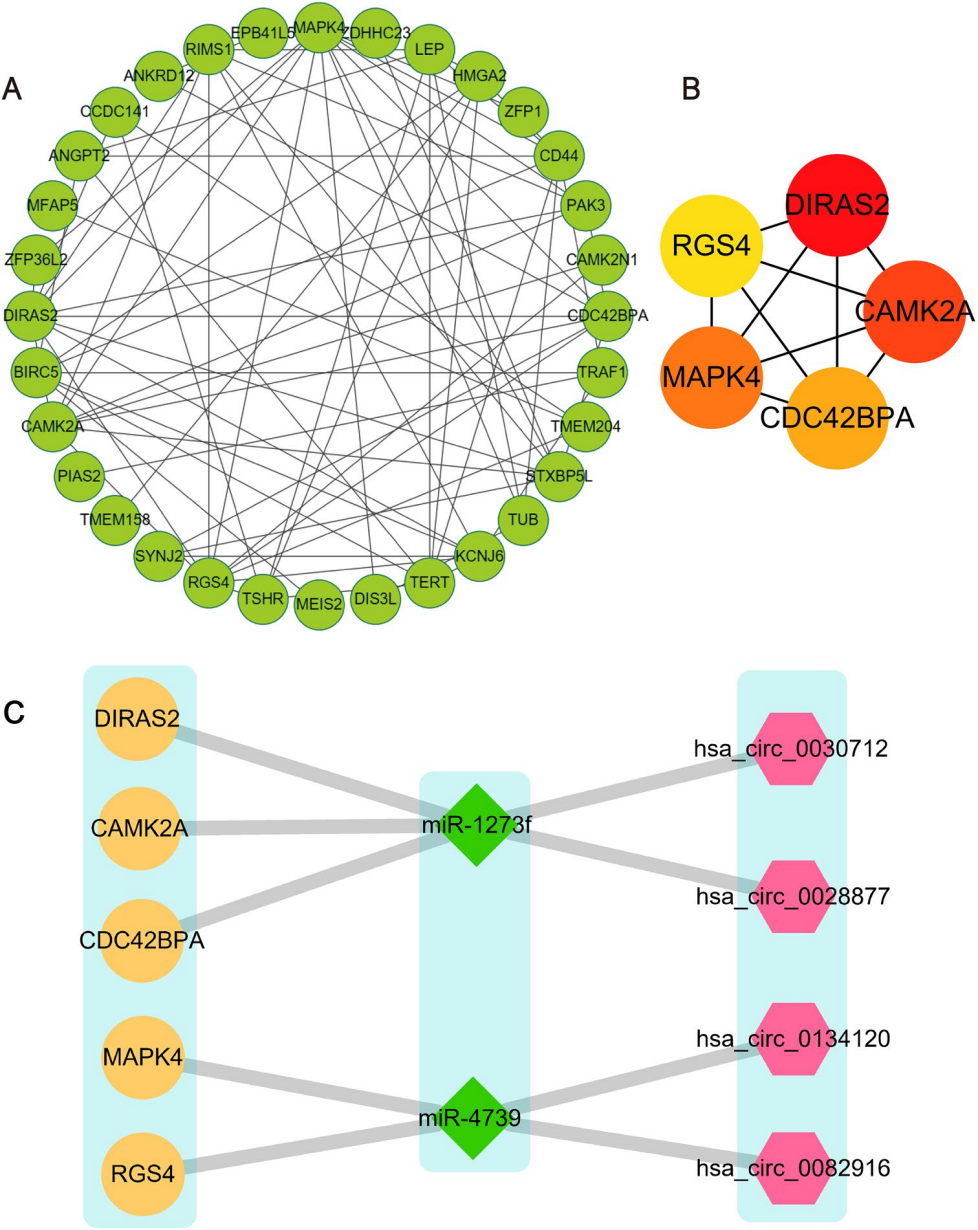
PPI network establishment and hub genes identification of the target genes. (**A**) PPI network of the target genes. (**B**) Identification of 5 hub genes from the PPI network with the Cytohubba algorithm. (**C**) CircRNA–miRNA–hub gene subnetwork. *PPI* protein–protein interaction.

To explore the interactions between proteins encoded by the target genes, a protein–protein interaction (PPI) network was built. In brief, after removing nodes not linked to any other nodes, 32 nodes and 74 edges were mapped in the PPI network (Fig. 8A). The Cytohubba app in Cytoscape software was used to identify hub genes that are more important in the PPI network and a significant module containing 5 nodes (DIRAS2, CAMK2A, MAPK4, CDC42BPA, and RGS4) and 10 edges was identified (Fig. 8B). Besides, it's worth noting that the CAMK2A and RGS4 in these hub genes were also significantly different in the RNA sequencing results.

Then, a circRNA–miRNA–hub gene subnetwork based on the five hub genes was constructed for further study (Fig. 8C). The binding sites between the target circRNAs and the target miRNAs predicted by circMir software were shown in Supplement Fig. 1 and Table 3, and the binding sites between the target miRNAs and the hub genes were shown in Table 4. We also found that hsa_circR_0134120 had the most binding sites to its target miRNAs.

**Table 3:**
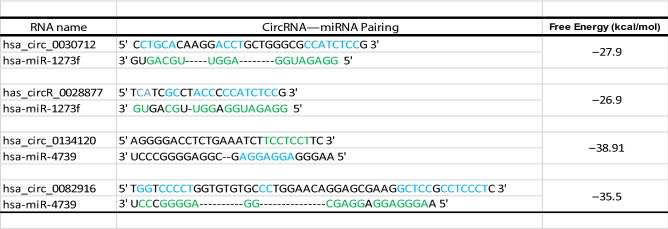
Predicted circRNA–miRNA binding sites and activity.

The blue and green bases represent binding sites between miRNAs and circRNAs.

**Table 4:**
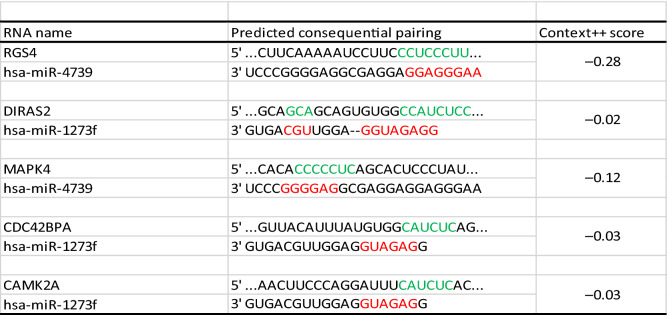
Predicted hub gene–miRNA binding sites and activity.

The red and green bases represent binding sites between miRNAs and mRNAs.

### qRT-PCR analysis of relative target RNAs

**Figure 9:**
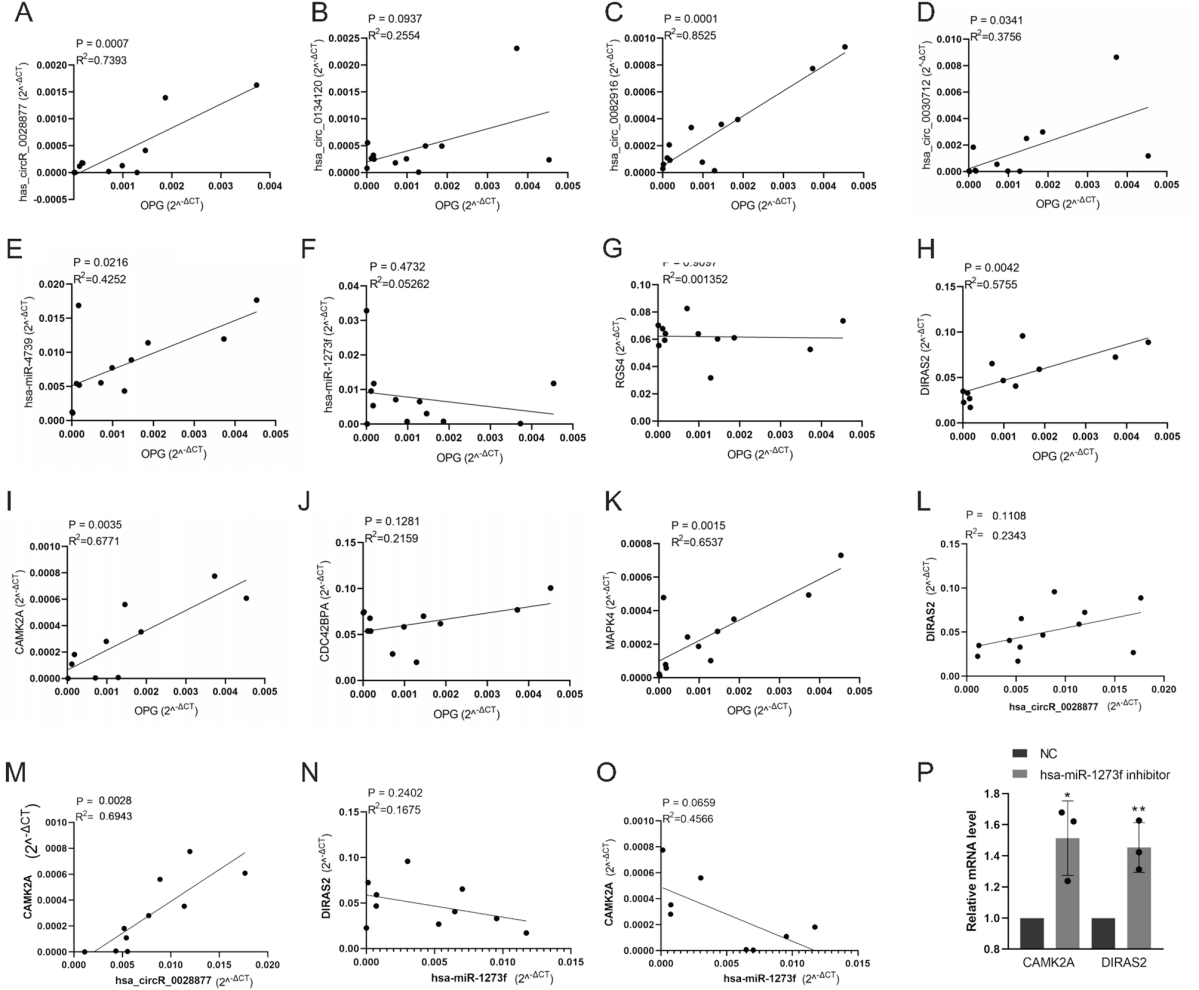
qRT-PCR correlation analysis of circRNAs, miRNAs, and mRNAs in the ceRNA sub-network with osteogenesis indicator OPG. (**A**–**D**) The correlation of circRNAs with OPG. (**E**, **F**) The correlation of miRNAs with OPG. (**G**–**K**) The correlation of mRNAs with OPG. (**L**) hsa_circR_0028877 expression positively correlated with DIRAS2. (**M**) hsa_circR_0028877 expression positively correlated with CAMK2A. (**N**) hsa-miR-1273f expression negatively correlated with DIRAS2. (**O**) hsa-miR-1273f expression negatively correlated with CAMK2A. (**P**) Inhibition of hsa-miR-1273f with an inhibitor in human BMSCs cells presents up-regulation of CAMK2A and DIRAS2.

To further examine the relationships between relative target RNAs and osteogenesis, we analyzed the correlations between the target circRNAs, target miRNAs, and target mRNAs in the ceRNA sub-network and the osteogenesis indicator OPG, in the same 12 cases of human bone tissues. As shown in Fig. 9A–K, the expression of hsa_circ_0028877, hsa_circ_0082916, hsa_circ_0030712, DIRAS2, CAMK2A, and MAPK4 was significantly correlated with osteogenesis.

Besides, with the correlation analysis of mRNA–circRNA, we judged that both the expressions of CAMK2A and DIRAS2 were positively correlated with the expression of hsa_circR_0028877 (Fig. 9L–M). As for the correlation analysis of miRNA-mRNA in the human bone tissues, we found that hsa-miR-1273f was negatively correlated with the target genes CAMK2A and DIRAS2 (Fig. 9N–O), and this conclusion was also tested by suppressing hsa-miR-1273f with an inhibitor in human BMSCs (Fig. 8P).

Eventually, the expression of hsa_circR_0028877/hsa-miR-1273f/CAMK2A and hsa_circR_0028877/hsa-miR-1273f/DIRAS2 was consistent with the characteristics of the ceRNA mechanism.

### Identification of the potential drugs and construction of drug-target gene interaction network

**Figure 10:**
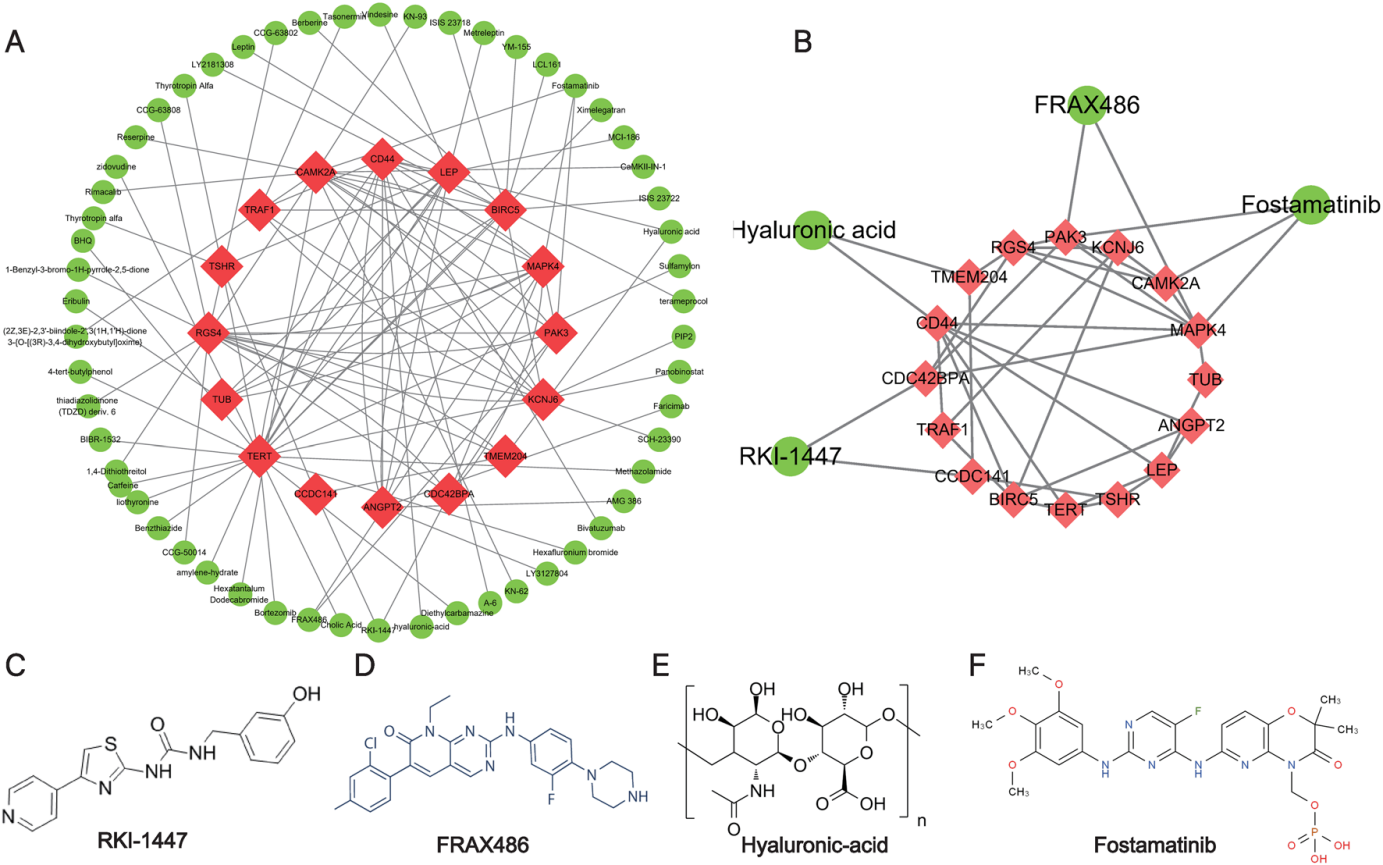
Identification of potential drugs and construction of drug-target gene interaction network and sub-network. (**A**) Drug-target gene interaction network. (**B**) Sub-network. The green circles represent the candidate drugs and the red rhombuses represent the proteins encoded by target genes. (**C**) Molecular structure of RKI-1447. (**D**) Molecular structure of FRAX486. (**E**) Molecular structure of Hyaluronic acid. (**F**) Molecular structure of Fostamatinib.

To find the potential drugs which might produce the curative effects of OP through the target genes, we uploaded 38 target genes from the ceRNA network to the Drugbank, TTD, and CLUE database and obtained 62 kinds of candidate compounds. Then the drug-target gene interaction network was constructed to visualize their interactions (Fig. 10A). Besides, as shown in Fig. 10B, to further screen the candidate compounds, the sub-network which contains the drugs that simultaneously target multiple target genes was constructed and 4 drugs were screened out: RKI-1447, FRAX486, Hyaluronic-acid, and Fostamatinib (Fig. 10C–F).

## Discussion

Osteoporosis (OP) is a systemic skeletal disease, which is related to advancing age and is characterized by the systemic destruction of bone mass and microarchitecture that results in fragility fractures^19^. With the increase of the aging population, the number of osteoporosis patients and particularly the postmenopausal osteoporosis patients are increasing rapidly^1^.

CircRNAs belong to the class of noncoding RNAs and were first discovered in humans in 1986^20^. CircRNAs have a complete closed‐loop structure in which the 3′ and 5′ ends are linked by a process termed “back-splicing” and are lacking a poly (A) tail. The closed‐loop structure makes circRNAs be resistant to exonucleases and thus are more stable than linear RNAs^21,22^. Besides, circRNAs have the characteristics of tissue, time, and disease specificity^21,23^. CircRNAs can act as miRNA sponges by binding to the specific miRNAs, thus relieving the inhibitory effects of miRNAs on their target genes and regulating the expression of target genes. This mechanism is known as the competitive endogenous RNA (ceRNA) mechanism^24^, which is a research hotspot at present. The abnormal expression of circRNAs was investigated in many diseases, such as colorectal cancer, liver cancer, and pancreatic ductal adenocarcinoma, etc.^25–27^, and was found to be involved in a variety of bone homeostases relative events, such as the osteogenesis differentiation and bone absorption^9,28^. However, studies of osteoporosis-associated circRNAs and ceRNA mechanisms were still rare at present.

In this study, to investigate the potential role of circRNAs in osteoporosis, we first constructed the circRNA–miRNA–mRNA (ceRNA) regulatory networks by using bioinformatics predictions combined with OP relative differential expression data from the GEO database. This network contained the interactions of 4 circRNAs, 5 miRNAs, and 38 mRNAs. In the ceRNA network, circRNAs act as miRNA sponges and regulate the expression of miRNA target transcripts. Here, we used a circRNA microarray dataset to get the DECs. 10 OP-related circRNAs were found and none of the functions of these DECs had been studied, thus the study of these circRNAs might reveal some new therapeutic targets.

To further elucidate the mechanism of the ceRNA network, we constructed a PPI network of target genes and screened out 5 hub genes. Among these hub genes, Xia Yi et.al has indicated that CAMK2A is involved in the regulation of adipogenesis and osteoblast genesis from human bone marrow mesenchymal stem cells^29^. Heirani-Tabasi A et.al have indicated that MAPK4 participates in the migration of adipose-derived mesenchymal stem cells under hypoxia-mimicking agents^30^. Li et al. have indicated that RGS4 protects against osteoporosis through the LINC_00370-miR_222_3p-RGS4 axis. However, the links of these genes with circRNAs have not yet been explored. Then, in order to pick out a few more important ceRNA networks, a sub-ceRNA network launch around the hub genes was constructed and 10 circRNA–miRNA–mRNA axes were identified. Besides, RNA-seq analysis verification of the target genes indicated that the expression of MFAP5, CAMK2A, and RGS4 was significantly changed in the OVX rats and is closely associated with osteoporosis. What’s more, the qRT-PCR analysis was also employed to verify these networks. The qRT-PCR analysis revealed that the expression of hsa_circ_0028877, hsa_circ_0082916, hsa_circ_0030712, DIRAS2, CAMK2A, and MAPK4 was significantly correlated with osteogenesis, and the hsa_circR_0028877-hsa-miR-1273f-CAMK2A axis and hsa_circR_0028877-hsa-miR-1273f-DIRAS2 axis were two potential OP related ceRNA mechanisms. However, although the expression level of hsa_circ_0134120 didn’t show a significant difference in the qRT-PCR results, there were many target miRNAs bound to hsa_circ_0134120 predicted by the circMir software (Supplement Fig. 1), thus this circRNA may be also involved in the development of OP.

In the enrichment analysis, the target genes were shown to be mainly involved in the MAPK pathway, cellular response to hormone, regulation of oxidoreductase activity, etc. Among them, the MAPK pathway was a classical osteogenesis-related pathway^10^, which could influence the differentiation of human bone marrow stromal cells into osteoblasts^31^, and the qRT-PCR analysis of the mRNA changes in MAPK’s expression level also demonstrated the correlation between this pathway and OP. Many types of hormones have been proved closely related to osteoporosis, such as estrogen and thyroid hormones; Estrogen could reduce bone resorption in women and promote osteoblast osteogenesis through Wnt/ERα and β-catenin pathway^32^, while thyroid hormone could improve osteoblast differentiation and activity^11^. As for the regulation of oxidoreductase activity, studies have shown that osteoblast ferroptosis-induced osteoporosis was associated with the oxidoreductase relative pathway^33^. Consistent with the above results, our results further proved that the above pathways and the related genes were closely related to osteoporosis. The GO and KEGG analysis of the target miRNAs showed that they were enriched in the ‘PI3K-Akt signaling pathway’, ‘Vitamin digestion and absorption’, ‘Thyroid hormone signaling pathway’, and ‘cell death’, and these items were extensively involved in osteoporosis^11,34–36^. On account of the lack of direct enrichment analysis methods for circRNA, the GO and KEGG analyses of the host genes were performed to further annotate the biological functions of the differentially expressed circRNAs indirectly. Interestingly, the host genes were enriched in the cadherin binding, rRNA methyltransferase activity, and cellular response to oxidized low-density lipoprotein pathway. All these pathways have been reported to be associated with osteoporosis^13,15,37^. These data indicated that the mRNAs, miRNAs, and circRNAs in our ceRNA network were likely to play important roles in OP, thus the ceRNA network might participate in the genesis and development of OP.

To make the most of the ceRNA network, we used the target genes to explore the potential compounds or drugs with latent therapeutic effects for osteoporosis and constructed a drug-protein network. By using Drugbank, TTD, and CLUE database, four potential chemicals were screened out, including RKI-1447, FRAX486, Hyaluronic acid, and Fostamatinib. RKI-1447 is a kind of Rho-kinase inhibitor with anti-invasive and antitumor activities in breast cancer^38^. FRAX486 is a kind of P21-activated kinase inhibitor and was reported to inhibit the growth of prostate stromal cells^39^. Hyaluronic acid, a main extracellular matrix component of articular cartilage, has been proved to promote the chondrogenic differentiation of human amniotic mesenchymal stem cells (hAMSCs). Fostamatinib is the first spleen tyrosine kinase (Syk) inhibitor approved for the treatment of chronic immune thrombocytopenia (ITP) in adult patients. However, no study has reported their effects on osteoporosis. Further research is needed to verify their uses in the treatment of osteoporosis. The drug-protein network constructed in the present study might contribute to the further study of these chemicals.

However, there are several limitations of this study that should be considered. Firstly, our study was based on a range of online databases and bioinformatics analysis methods, and our method can't screen out miRNAs related to the ceRNA mechanisms but their own expression was not affected, which is the general situation in the ceRNA mechanisms. However, this analysis process could help to find out some of the miRNAs that are closer to osteoporosis, because all the ceRNA mechanism-related miRNAs in this study were screened out from the osteoporosis-related differentially expressed miRNAs. Secondly, the specific mechanisms in the ceRNA network, including the miRNA-mRNA interaction and circRNA–miRNA interaction, have not been verified by a series of molecular biology experiments, and further research is needed to be performed to confirm the conclusions in this study. Thirdly, when we look for osteoporosis-related publicly available high-throughput data, not all the data were from human bone tissues, and some of them were from plasma and some were from different species. This will also affect the accuracy of the data to some extent. Therefore, further specific experimental validation is needed to evaluate the conclusions.

## Conclusion

In conclusion, by employing a comprehensive strategy of bioinformatics analysis and experimental verification, we constructed a circRNA–miRNA–mRNA network and found that hsa_circ_0028877, hsa_circ_0082916, hsa_circ_0030712, DIRAS2, CAMK2A, and MAPK4 were significantly correlated with osteogenesis, and the axis of hsa_circR_0028877/hsa-miR-1273f/CAMK2A and hsa_circR_0028877/hsa-miR-1273f/DIRAS2 might function as ceRNA mechanisms to exert critical roles in osteoporosis. In addition, 4 bioactive chemicals (RKI-1447, FRAX486, Hyaluronic acid, and Fostamatinib) were determined as potential therapeutic agents for osteoporosis.

## Methods

### Microarray data mining and identification of DEGs, DEMs, and DECs

The microarray data used in this study were screened out from the GEO database, a functional genomic database of NCBI (http://www.ncbi.nlm.nih.gov/geo). There were three mRNA expression profiles (GSE35956, GSE116925, and GSE156508), two miRNA expression profiles (GSE74209 and GSE93883), and one circRNA expression profile (GSE161361) obtained from the GEO database and then the online tool GEO2R (https://www.ncbi.nlm.nih.gov/geo/geo2r/) was used to dig out the differentially expressed RNAs. GEO2R allows users to compare two or more groups of samples in a GEO dataset in order to identify genes that are differentially expressed across experimental conditions.

After the data processing of the GEO datasets by GEO2R, the selected RNAs were analyzed with the website of the Venn diagram tool (http://bioinformatics.psb.ugent.be/webtools/Venn/). Then the sum of the intersections between every two filtered data sets of the mRNA datasets was defined as the differentially expressed genes (DEGs); the intersections between the two filtered data sets of miRNA datasets were defined as the differentially expressed miRNAs (DEMs); and the analysis result of the circRNA expression profiles by GEO2R was defined as the differentially expressed circRNAs (DECs, also defined as the target circRNAs).

### Identification of the target miRNAs and the target genes

By using the online tool Venn diagram (http://bioinformatics.psb.ugent.be/webtools/Venn/), overlapping miRNAs between the predicted target miRNAs of DECs (Predicted target miRNAs were contained in the GSE161361 dataset) and the DEMs were defined as the target miRNAs.

miRWalk (http://mirwalk.umm.uni-heidelberg.de/) is a frequently-used open-source platform providing an intuitive interface that generates predicted and validated miRNA-binding sites of known genes^40^ and TargetScan (http://www.targetscan.org/vert_71/) can predict biological targets of miRNAs by searching for the presence of conserved 8mer, 7mer, and 6mer sites that match the seed region of each miRNA^41^. The miRWalk and the TargetScan website were used to obtain the predicted target mRNAs of DEMs which could bind with the target miRNAs. Overlapping between the predicted target mRNAs of DEMs and the DEGs was defined as the target genes. Besides, the significance of expression differences of the target genes was verified by RNA-seq.

### Construction of the ceRNA network

Based on the miRNA-mRNA and circRNA–miRNA interactions, the circRNA–miRNA–mRNA regulatory network was established and visualized by Cytoscape software (Version 3.7.2), a network biology analysis and visualization tool for integrating biomolecular interaction networks with high-throughput gene expression data and other molecular state information. In addition, only the target miRNAs that were predicted to combine with both the DECs and the target genes were selected for the establishment of the ceRNA network. Besides, the heat map of the target genes in the ceRNA network was constructed by Heml 1.0.3.7 software (http://hemi.biocuckoo.org/down.php).

### PPI network establishment and hub genes identification of the target genes

To analyze the target genes selected in the ceRNA network, a PPI network was established by the STRING (https://string-db.org), a powerful visualization and customization site for protein interaction analysis, and then re-visualized by the Cytoscape software (version 3.7.2). Then, one plugin of the Cytoscape software named Cytohubba used to identify hub objects and sub-networks from complex interactome^42^, was employed to recognize the highly interacted hub genes by calculating the degree, betweenness centrality, and closeness centrality of mRNAs in the PPI network. Overlapping and top‐ranking genes among the three calculation methods were defined as the hub genes. Besides, the hub gene relative ceRNA network was filtered out from the original ceRNA network.

### Enrichment analysis of target genes, target miRNAs, and DECs

Metascape online tool (http://metascape.org) and the R package ggplot2 (version 3.3.3) and clusterProfiler were used for the GO/KEGG enrichment analysis of the target genes. Metascape is a web-based portal designed to provide a comprehensive gene list annotation and analysis resource for experimental biologists. Besides, the clusterProfiler package was used to obtain sufficient items, which can crawl the gene-phenotypic relationship data in real-time to keep the enrichment analysis results up to date, and the results were visualized with the R package ggplot2.

The GO annotation and KEGG pathway analyses of the target miRNAs were conducted by the mirPath v.3 tool on the DIANA TOOLS website (http://snf-515788.vm.okeanos.grnet.gr/). mirPath can utilize predicted miRNA targets (in CDS or 3′-UTR regions) provided by the DIANA-microT-CDS algorithm or experimentally validated miRNA interactions derived from DIANA-TarBase. These interactions (predicted/validated) can be subsequently combined with sophisticated merging and meta-analysis algorithms to obtain miRNAs’ targets^43^.

As for the enrichment analysis of DECs, the host genes of DECs were chosen as the research objects for the enrichment analysis with the R packages ggplot2 and clusterProfiler.

### Prediction of OP-associated drugs and extraction of relevant target genes in the ceRNA network

Drugbank (https://go.drugbank.com/), TTD (Therapeutic target database, http://db.idrblab.net/ttd/)^44^, and CLUE database (Connectivity Map Linked User Environment, https://clue.io/) were selected to predict the target genes and the related potential drug molecules for OP treatment. The predicted results and related target genes were imported into the Cytoscape software and mapped into a drug-gene network. To further screen out more significant drug molecules, the drugs that simultaneously target multiple target genes were singled out.

### Animals

Twenty female Wistar rats were obtained from the Experimental Animal Center of Dalian Medical University (Dalian, China). All animal studies complied with the Guidelines for the Care and Use of Laboratory Animals of the National Institutes of Health and all experimental protocols in the current study and were approved by the institutional animal care committee of Dalian Medical University (Dalian, China; Certificate of Conformity: No. SCXK 2018-0007), and all animal experiments were performed following ARRIVE. The rats were housed in pathogen-free facilities under a 12/12 h light/dark cycle and the temperature during the experiment was maintained at around 23 ± 2 °C. The rats were fed with the standard laboratory diet and drink deionized water freely. Female rats were undergoing either bilateral oophorectomy or bilateral sham operation after pentobarbital sodium anesthesia. One week later, the surviving rats were divided into the Oophorectomy group (OVX, n = 10) and the Control group (SHAM, n = 1 0). After 8 weeks of normal feeding, all animals were anesthetized with pentobarbital sodium and the femurs were obtained for the next experiment.

### RNA isolation and RNA sequencing

Total RNA was isolated from the femurs of 6 rats (3 with ovariectomy and 3 healthy controls) using TRIzol reagent (Sigma, St. Louis, USA), following the manufacturer’s protocol. The femur was ground after freezing with liquid nitrogen in the absence of RNA enzymes. Then we isolated total RNAs from each sample and treated them with DNase I to degrade any potential DNA contamination. The quality and quantity of RNA were tested on a Nanodrop ND-2000 spectrophotometer (Thermo Scientific). An equivalent of 5 mg RNA was utilized as sequencing samples and the RNA-seq was performed on the Illumina HiSeq™ Xten platform by IGENE-BOOK Biotechnology Ltd. (Wuhan, www.igenebook.com). R package limma16 was carried out to normalize the expression data and calculate the differential expression. The miRNAs with fold change > 2 and P < 0.05 were differentially expressed. The accession of our project is AJRS2210325019 and the raw sequencing data has been uploaded to the GEO database (GSE201674).

### Clinical samples and qRT-PCR analysis

The cancellous bone tissue was gathered from 12 patients who received surgical treatment and were frozen and stored in liquid nitrogen. The written informed consent of each subject was obtained for this experiment and this study was approved by the ethics institute of the First Affiliated Hospital of Dalian Medical University (YJ-KY-FB-2021-22) and information about the patient's age, gender, and health status was recorded. The collected cancellous bone tissue was analyzed by qRT-PCR (Quantitative Real-time PCR) analysis.

The expression of potential circRNAs, miRNAs, and genes in these clinical samples was detected by the method of qRT-PCR. Reverse transcription of cDNA was conducted using EasyScript® One-Step gDNA Removal and cDNA Synthesis SuperMix reagent kit (Transgen, Beijing, China). Quantitative Real-Time PCR was performed by TransStart® Top Green qPCR SuperMix (+ Dye II) (Transgen, Beijing, China) on a qPCR instrument (Bio-rad, USA). The relative level of the target gene was calculated using the 2^−∆CT^ method. The Primers used in this study were synthesized and purchased from RiboBio Co. Ltd (Guangzhou, China), and the sequences of primers were shown in Table 5.

**Table 5:** Primers used in the RT-qPCR.

Reverse primer	RT primer
AGCATAGTGCAAGGTTGTAATCC	Oligo (dT)
CCTCTGAGATGCTGTCATGTAGT	Oligo (dT)
GATCTGGAGACTCACGGTCCT	Oligo (dT)
GTTGTGGGAAGAATTGTGTTCAC	Oligo (dT)
GACGATGTTGTCGTGGTCCA	Oligo (dT)
CCAGTGCAGGGTCCGAGGT	GTCGTATCCAGTGCAGGGTCCGAGGTATTCGCACTGGATACGCACTGC
CCAGTGCAGGGTCCGAGGT	GTCGTATCCAGTGCAGGGTCCGAGGTATTCGCACTGGATACGAGGGCC
TCTTCCACATGCGAGTTTAAGT	Random primer
TGTGTTTTGGTATGCAGGGC	Random primer
GGCTCCCCAATTTCTCACCT	Random primer
AGTCCGCCATCTTCCGAAG	Random primer

To avoid interference of homologous linear RNAs, the splice junction overlapping divergent primers (Sjod Primers) were used during the detection of circRNAs. As for the detection of miRNAs, in order to obtain the accurate expression level, we used the stem-loop reverse primers to reverse the miRNA to cDNA, and then a pair of amplification primers were used to detect the Ct (Cq) values.

### Statistical analysis

Expression correlation for RNA-RNA interactions in the qRT-PCR analysis part was performed by Person correlation coefficient using GraphPad Prism software (version 7). Results with P-value < 0.05 were considered statistically significant.

### Ethics statement

All the tissue samples were collected with written informed consent following the Declaration of Helsinki and with the approval of the First Affiliated Hospital of Dalian Medical University.

## Supplementary Information

Supplementary Legends.

Supplementary Figure S1.

Supplementary Figure S2.

Supplementary Figure S3.

## Data Availability

The datasets GSE74209, GSE161361, GSE116925, GSE35956, and GSE156508 for this study can be found in the Gene Expression Omnibus (https://www.ncbi.nlm.nih.gov/geo/). The sequencing data of this study can be found in the supplementary material.
