# Supplementary material for: The integrative analysis of competitive endogenous RNA regulatory networks in osteoporosis
Source: Sci Rep. 2022 Jun 9;12:9549. doi: 10.1038/s41598-022-13791-0 (PMC9184474; doi:10.1038/s41598-022-13791-0)
Supplement: Supplementary file 1 — Supplementary Legends. [file 41598_2022_13791_MOESM1_ESM.docx]

**Supplement figure 1 Predicted binding sites between circRNA and miRNA.** The points on the ring represent reverse splicing sites.

**Supplement figure 2 Enrichment analysis of RNA-seq results. (**A) (B) GO and KEGG analysis of down-regulated genes in the OVX rats. (C) (D) GO and KEGG analysis of up-regulated genes in the OVX rats.

**Supplement figure 3 The overlap** **between 104 DEGs and RNA-seq data.**
