# Supplementary figures and images for: The integrative analysis of competitive endogenous RNA regulatory networks in osteoporosis
Source: Sci Rep. 2022 Jun 9;12:9549. doi: 10.1038/s41598-022-13791-0 (PMC9184474; doi:10.1038/s41598-022-13791-0)

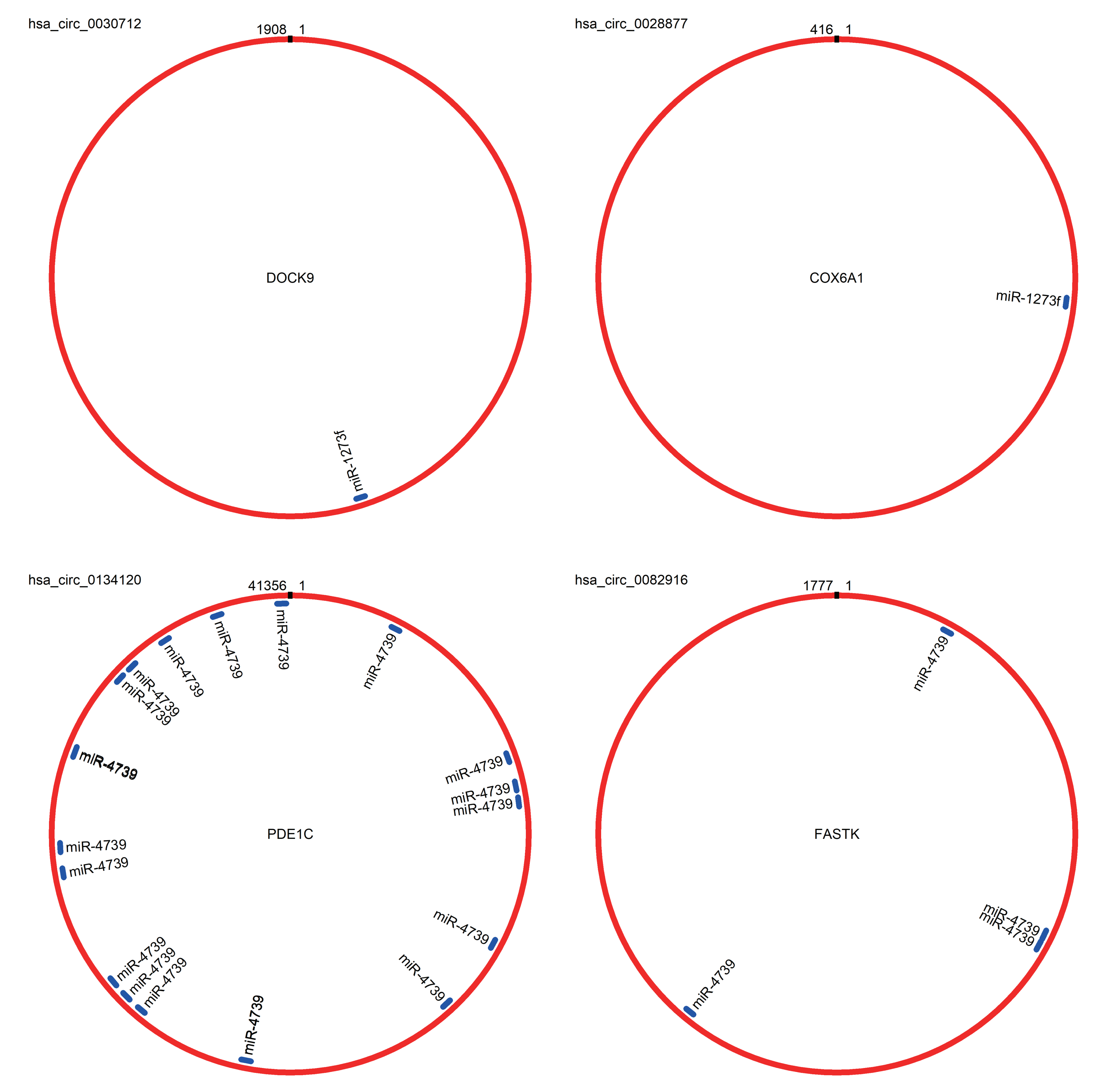

Supplement: Supplementary file 2 — Supplementary Figure S1. [file 41598_2022_13791_MOESM2_ESM.tif]

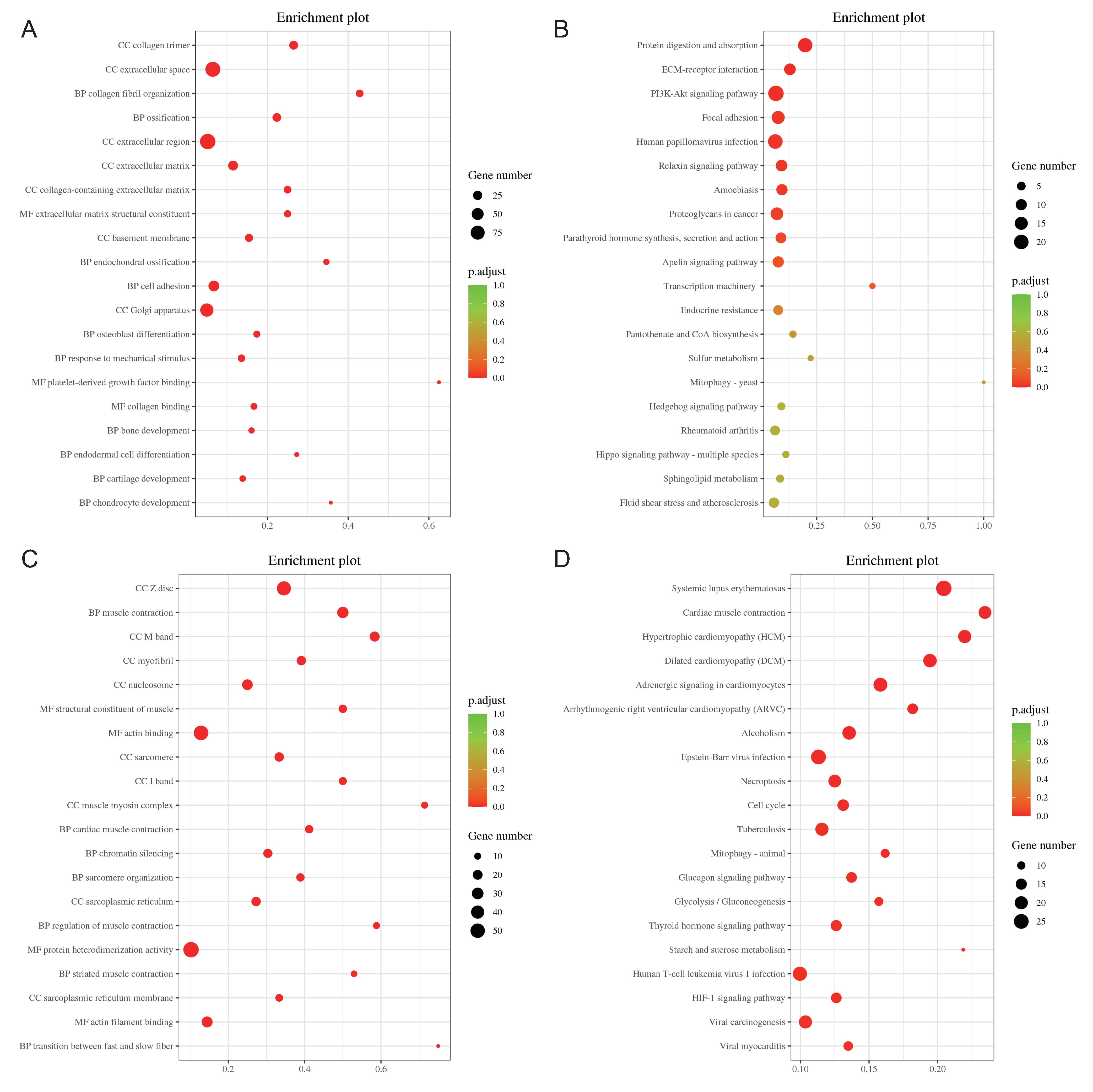

Supplement: Supplementary file 3 — Supplementary Figure S2. [file 41598_2022_13791_MOESM3_ESM.tif]

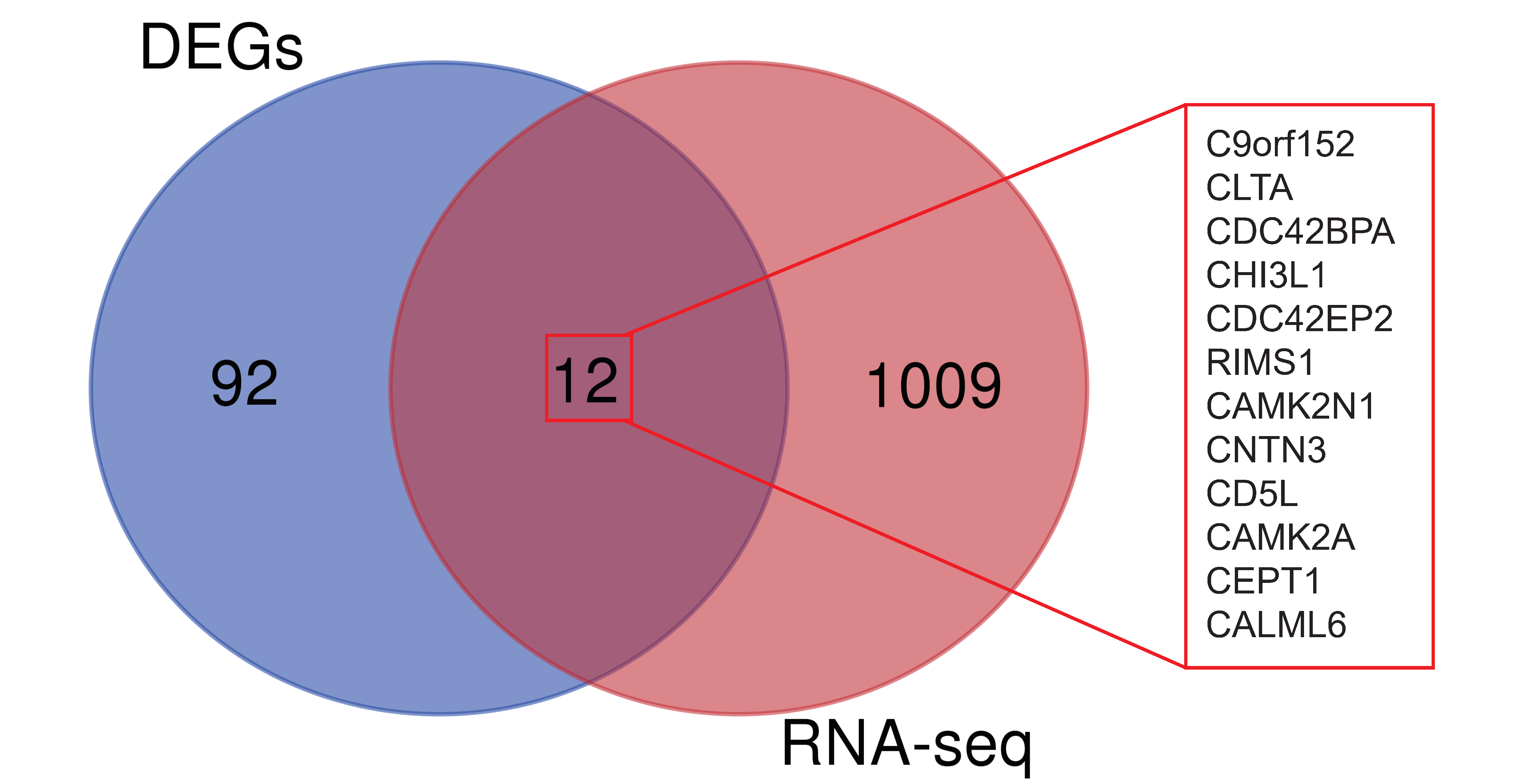

Supplement: Supplementary file 4 — Supplementary Figure S3. [file 41598_2022_13791_MOESM4_ESM.tiff]
